# Serum HMGB1 Serves as a Novel Laboratory Indicator Reflecting Disease Activity and Treatment Response in Ankylosing Spondylitis Patients

**DOI:** 10.1155/2016/6537248

**Published:** 2016-10-05

**Authors:** Chenqiong Wang, Ye Miao, Xuefen Wu, Yishu Huang, Mengchen Sun, Yingzi Zhu, Fang Zheng, Wei Sun, Lingli Dong

**Affiliations:** ^1^Department of Rheumatology and Immunology, Tongji Hospital, Tongji Medical College, Huazhong University of Science and Technology, 1095th Jiefang Avenue, Wuhan, Hubei 430000, China; ^2^Department of Immunology, Tongji Medical College, Huazhong University of Science and Technology, Wuhan 430000, China; ^3^Department of Stomatology, Union Hospital, Tongji Medical College, Huazhong University of Science and Technology, Wuhan 430000, China

## Abstract

*Objective*. High mobility group box 1 (HMGB1) is a late inflammatory factor participating in the pathogenesis of various autoimmune and inflammatory diseases. In the current study, we analyzed the association between serum levels of HMGB1 and clinical features of AS patients before and during treatment.* Methods*. Serum HMGB1 was detected in 147 AS patients and 61 healthy controls using ELISA. We evaluated the association between HMGB1 and extra-articular manifestations as well as disease severity indices. Among these AS patients, 41 patients received close follow-up at 1, 3, and 6 months after treatment. This group comprised 25 patients treated with anti-TNF-*α* biologics and 16 patients receiving oral NSAIDs plus sulfasalazine.* Results*. The serum HMGB1 of AS patients was significantly higher than in healthy controls and positively correlated with BASDAI, BASFI, ASDAS-ESR, ASDAS-CRP, ESR, and CRP, but not with HLA-B27, anterior uveitis, and recurrent diarrhea. There was no significant difference between patients with radiographic damage of hip joints and those without. We observed that serum HMGB1 paralleled disease activity after treatment.* Conclusion*. Serum level of HMGB1 is higher in AS patients, and to some extent, HMGB1 can reflect the activity of AS and be used as a laboratory indicator to reflect the therapeutic response.

## 1. Introduction

Ankylosing spondylitis (AS) is a chronic inflammatory disease, characterized by the inflammation of sacroiliac joints and the spine, which mainly affects young males including disability and decreased quality of life [1]. Apart from the involvement of axial joints, extra-articular manifestations such as inflammatory bowel disease (IBD) and anterior uveitis are relatively common. Although the pathogenesis of AS is not completely unclear, it is widely accepted as the interaction between environmental factors and genetic risk factors, which has a strong association with human leukocyte antigen-B27 (HLA-B27) [2, 3]. However, recent genome-wide association studies (GWASs) have identified that some single nucleotide polymorphisms (SNPs) were associated with AS. Many of these gene loci assemble in dissimilar immunoregulatory pathways [4], which are related to NF-*κ*B signaling, IL-23 pathway, and T cell phenotype as well as antigen presentation [5, 6]. Inflammation and new bone formation are considered as the critical factors of AS, within the genetically susceptible individuals. Abnormal inflammation and immunity are also considered as major etiological factors, involving the upregulation of the inflammatory cytokines including tumor necrosis factor-alpha (TNF-*α*), IL-23, and IL-17 [7], and anti-IL17 agents showed similar therapeutic efficacy to TNF blockers in clinical trials [8]. DAMP (damage-associated molecular pattern) formed by exogenous microbial PAMPs (pathogen-associated molecular patterns) and endogenous alarmins play important roles in inflammatory processes. Previous studies have revealed that PAMPs, such as intra-articular microbial antigens, may be involved in the development of syndesmophytes and bamboo spine in AS [9]. As a typical alarmin, high mobility group box 1 (HMGB1) acts as an inflammatory mediator to participate in the pathogenesis of many kinds of autoimmune and inflammatory diseases. However, there exists scarce research pertaining to HMGB1 and AS, which prompted us to investigate the association between HMGB1 and AS.

HMGB1 is a highly conserved nuclear nonhistone protein, which has been previously characterized as DAMP involved in inflammatory processes [10]. HMGB1 is distributed in the nuclei of almost all eukaryotic cells and can shuttle from nucleus to cytoplasm depending on the acetylation of lysine residues within the nuclear localization signal domains (NLS) [11]. AS a typical alarmin, HMGB1 acts as a proinflammatory cytokine when released to the extracellular environment actively or passively and binds to receptors such as TLR2, TLR4, and RAGE, which is short for receptor for advanced glycation end products. HMGB1-mediated signaling pathway can promote the production of TNF-*α*, IL-1*β*, and IL-6, which are all involved in the pathology of AS [12, 13]. Furthermore, HMGB1 can be chemotactic to mesenchymal stromal cells, monocytes, and osteoblasts, which leads to ectopic enchondral bone formation [9]. When released by apoptotic osteoblasts in vitro, HMGB1 plays a role as a bone-active cytokine, which can promote the activation and differentiation of osteoclasts leading to bone destruction [14, 15]. Taken together these observations indicate that HMGB1 may play a role in the pathological progression of AS.

However, little research has focused on the association between HMGB1 and AS. Results from Oktayoglu et al. [16] and Chen et al. [17] indicated that high expression of HMGB1 may be involved in the pathogenesis of AS, but all consequent studies involved limited numbers of cases, lacking in consistent observations including the changes of HMGB1 during disease progression and different treatment strategies in AS patients. In our current study, we detected the serum levels of HMGB1 in 147 AS patients compared with 61 healthy controls and analyzed the relationship between HMGB1 and disease activity. Furthermore, we examined the trend of HMGB1 in AS patients during six-month follow-up, including 25 patients who were treated by anti-TNF-*α* block therapy and 16 patients treated with NSAIDs and SASP.

## 2. Materials and Methods

### 2.1. Patient Characteristics and Samples

147 AS patients (23 women, 124 men; age range, 15~56 y; mean age, 30.28 ± 9.85 y) were recruited from outpatients of the Department of Rheumatology at Tongji Hospital of Tongji Medical College, Huazhong University of Science & Technology (HUST), between January 2015 and September 2015. The enrolled patients fulfilled the modified New York criteria [18, 19] and were without treatment during the previous 3 months. The exclusion criteria of this study were patients with a history of cancer, recurrent infections, or other types of rheumatic diseases. All patients filled in a questionnaire, which was used to record the clinical information as well as disease severity index, including name, age, sex, Bath AS Disease Activity Index (BASDAI), AS Disease Activity Score (ASDAS), and Bath AS Functional Index (BASFI). We also obtained laboratory assessments of all participants, including C-reactive protein (CRP) and erythrocyte sedimentation rate (ESR). The detection of ESR used the Westergren method, while CRP was measured by immunonephelometry using CRP reagents (BioSystems SA, Spain). The normal range of CRP was defined as 0~10 mg/dL in Tongji Hospital. 135 enrolled AS patients were tested with HLA-B27, while 101 patients received CT or MRI of sacroiliac joints as well as hip joints in the last 2 months. Among all AS patients, only 41 patients received close observation and follow-up for six months. These patients were divided into 2 groups, including an anti-TNF-*α* therapy group composed of 25 patients who were given adalimumab (*n* = 12), etanercept (*n* = 8), and infliximab (*n* = 5) for a total period of 6 months. The other group was treated with nonsteroidal anti-inflammatory drugs (NSAIDs) for one month followed by sulfasalazine (SASP) 2-3 g/d for the remainder of the study. Moreover, we also recruited 61 age- and sex-matched healthy volunteers as controls, without a history of cancers, recurrent episodes of infections, or family history of AS. This study was approved by the ethics committee of Tongji Hospital of Tongji Medical College, HUST (IRB ID: TJ-C20141213), and all patients registered their informed consent to participate in this study.

### 2.2. Samples and Determination

Peripheral blood was obtained from all enrolled outpatients and the follow-up patients as well as the healthy volunteers. The blood samples were centrifuged at 4000 rpm for 5 minutes. Serum was stored at −80°C. Serum levels of HMGB1 were measured with the commercially available enzyme linked immunosorbent assay (ELISA) kit (Uscn Life Science Inc., Wuhan, China) according to the instruction manual.

### 2.3. Statistical Analysis

Database management and statistical analyses were performed using SPSS 19.0 (SPSS, Chicago, IL, USA). The results of baseline and follow-up from different groups were compared with independent samples using Student's *t*-test and expressed as mean value ± standard deviation (SD). Correlations between variables were conducted using Spearman's rank correlation test and *P* values < 0.05 were set as a statistically significant difference.

## 3. Results

Table 1 shows baseline characteristics of 147 AS patients and 61 healthy controls in our study. The level of serum HMGB1 in AS patients was significantly higher than the healthy controls (HMGB1: 106.81 ± 30.87 ng/mL versus 27.68 ± 17.95 ng/mL, *P* < 0.001). We assessed the correlation between HMGB1 and other indices during this study, including the baseline and the follow-up time point. The HMGB1 level was positively correlated with BASDAI (*r* = 0.304), BASFI (*r* = 0.184), ASDAS-ESR (*r* = 0.275), ASDAS-CRP (*r* = 0.251), CRP (*r* = 0.132), and ESR (*r* = 0.162); results are shown in Table 2.

The 101 AS patients receiving CT or MRI of sacroiliac joints as well as hip joints in the last 2 months were divided into two groups based on whether hip joints were involved. Pathological changes of hip joints were identified according to CT or MRI diagnosis and the minimum inclusion criterion was bone erosion. Serum HMGB1 showed no statistically significant difference between the two groups (HMGB1 109.40 ± 36.23 ng/mL versus 99.94 ± 25.31 ng/mL), while the BASDAI, BASFI, ASDAS-ESR, and ASDAS-CRP scores in the AS patients with hip joint involvement were all significantly higher than those without pathological changes (BASDAI: 3.85 ± 1.54 versus 3.13 ± 1.56, *P* < 0.05; BASFI: 2.88 ± 1.88 versus 2.01 ± 1.50, *P* < 0.05; ASDAS-ESR: 3.15 ± 1.48 versus 2.55 ± 0.99, *P* < 0.05; ASDAS-CRP: 3.23 ± 1.47 versus 2.64 ± 1.09, *P* < 0.05).

In 135 AS patients HLA-B27 was detected (107 positive versus 28 negative), and there was no significant statistical difference between the positive and the negative group (108.71 ± 30.65 ng/mL versus 98.08 ± 28.92 ng/mL). The serum HMGB1 of the enrolled AS patients who had a history of recurrent episodes of anterior uveitis (13 positive versus 134 negative) or diarrhea (13 positive versus 134 negative) was not significantly higher than in those without these symptoms (103.77 ± 44.36 ng/mL versus 107.11 ± 29.46 ng/mL, 93.14 ± 22.31 ng/mL versus 108.14 ± 31.32 ng/mL). Additionally, there were no statistically significant differences in the other indexes (BASDAI, BASFI, and ASDAS) between these two groups (data not shown).

**Figure 1:**
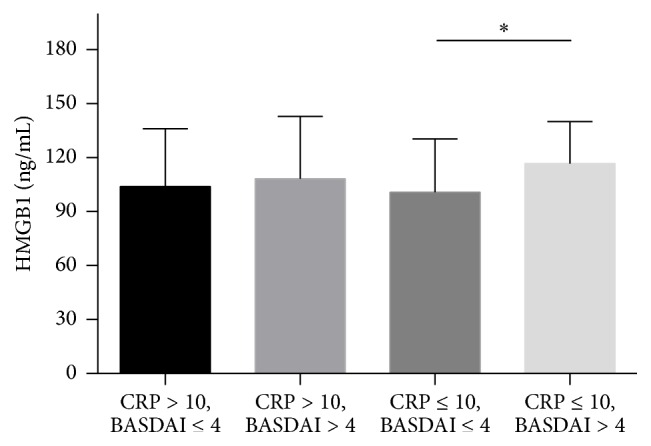
The comparison of serum HMGB1 in 4 groups of 147 AS patients, which were divided according to the value of CRP and BASDAI. Data include serum HMGB1 levels of 147 AS patients which were divided into 4 groups, defined as group 1 (CRP > 10 mg/dL + BASDAI ≤ 4, *n* = 36), group 2 (CRP > 10 mg/dL + BASDAI > 4, *n* = 40), group 3 (CRP ≤ 10 mg/dL + BASDAI ≤ 4, *n* = 51), and group 4 (CRP ≤ 10 mg/dL + BASDAI > 4, *n* = 20). BASDAI: Bath AS Disease Activity Index; CRP: C-reactive protein. ^*∗*^ *P* < 0.05.

In this study, the normal range of CRP was 0~10 mg/dL, while the patients with active disease were defined as a BASDAI of 4 [20–22]. 147 AS patients were divided into 4 groups defined as follows: CRP > 10 mg/dL + BASDAI ≤ 4 (*n* = 36), CRP > 10 mg/dL + BASDAI > 4 (*n* = 40), CRP ≤ 10 mg/dL + BASDAI ≤ 4 (*n* = 51), and CRP ≤ 10 mg/dL + BASDAI > 4 (*n* = 20). As reflected in Figure 1, despite the normal CRP value at baseline (CRP ≤ 10 mg/dL), serum HMGB1 of the BASDAI > 4 group was significantly higher than in the group with BASDAI ≤ 4 (116.80 ± 23.29 ng/mL versus 98.74 ± 33.16 ng/mL) as well as BASFI (2.73 ± 1.76 versus 1.32 ± 1.09), ASDAS-ESR (2.83 ± 0.66 versus 1.90 ± 0.59), and ASDAS-CRP (2.61 ± 0.73 versus 1.77 ± 0.64), which is not shown in Figure 1. Nevertheless, there was no statistical difference in HMGB1 between the other two groups with CRP > 10 mg/dL at baseline, regardless of whether BASDAI > 4.

In addition to this cross-sectional study, 41 enrolled patients were measured regularly during conducting a regular follow-up for a period of six months in our study. These AS patients were divided into two groups based on the therapeutic approach. Anti-TNF biologics treatment group included 25 patients and did not take oral medication during the six-month treatment period. The other group of 16 patients received accepted NSAIDs and SASP according to the recommended dosage during this period research. As shown in Table 3, the characteristics, including age, ESR, CRP, BASDAI, BASFI, ASDAS-CRP, and ASDAS-ESR, of the two treatment groups at baseline were very similar and had no statistically significant differences in the level of *P* = 0.05.

**Figure 2:**
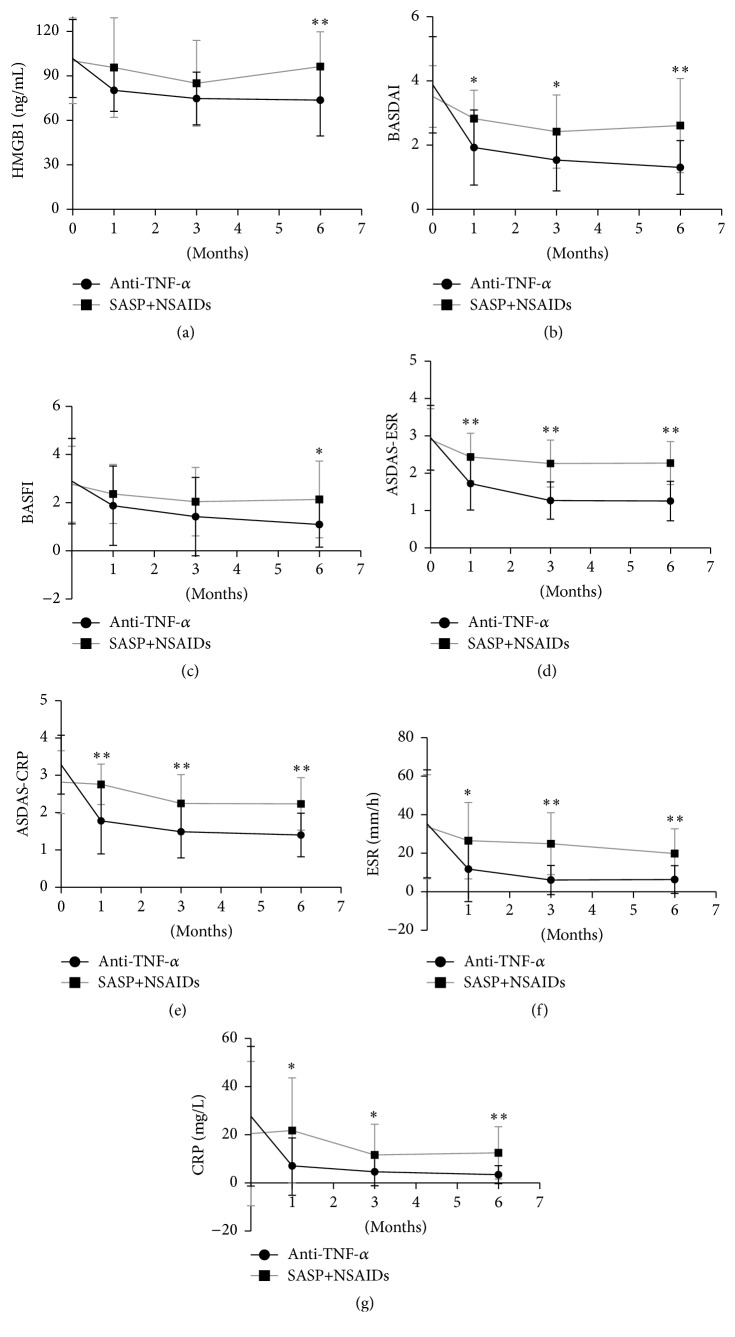
Changes in HMGB1 level and disease severity indices during follow-up. The black lines represent above indexes of AS patients treated with anti-TNF-a biologics and the gray lines represent patients treated with SASP and NSAIDs, during follow-up period. *∗* indicates *P* < 0.05; *∗∗* indicates *P* < 0.01.

Compared with the oral medication group, HMGB1 level and other indexes of the biologic group all decreased significantly relative to the baseline at three follow-up time points (Table 4 and Figure 2). It is worth noting that all indicators as well as HMGB1 decreased significantly in the first month and maintained a relatively steady downward trend for the next period of the study. Small fluctuations did not affect the downward trend throughout the 6 months of treatment. At the time point of 6 months during the treatment, HMGB1 of oral medicine group was significantly higher than biological group (96.39 ± 23.51 ng/mL versus 73.68 ± 24.20 ng/mL, *P* < 0.01). There also exist significantly statistical differences of the other indices at the three time points between the two groups, except for the BASFI (Figure 2(c)). And moreover, HMGB1 decreased by 20.57 ng/mL (20.21%, *P* < 0.05 versus baseline) but increased by 1.52 ng/mL (1.51%, *P* > 0.05 versus baseline) after 1 month in the anti-TNF and SASP + NSAIDs groups. Further, HMGB1 decreased by 25.35 ng/mL (24.91%, *P* < 0.05 versus baseline) and 12.97 ng/mL (12.93%, *P* < 0.05 versus baseline) at 3 months, respectively. At the end of the study, HMGB1 decreased by 28.10 ng/mL (27.60%, *P* < 0.05 versus baseline) while there was almost no statistical change compared with baseline in the oral medication group at 1 month and 6 months (Figure 2(a)). There was also a significant decline in BASDAI, BASFI, ASDAS-CRP, ASDAS-ESR, CRP, and ESR during the 6-month treatment period. This is further characterized by a particularly deep decline for the first month and maintained a downward trend during the six months of treatment. Scores for BASDAI, BASFI, ASDAS-ESR, and ASDAS-CRP decreased by 1.87 (45.95%, *P* < 0.05 versus baseline), 1.17 (37.26%, *P* < 0.05 versus baseline), 1.30 (44.07%, *P* < 0.05 versus baseline), and 1.57 (48.61%, *P* < 0.05 versus baseline) in the first month, respectively. Unlike the biological therapy group, except for the statistical reducing of BASDAI (16.19%, *P* < 0.05 versus baseline) and ASDAS-ESR (21.03%, *P* < 0.05 versus baseline) and at 1 month, there was no statistical decreasing of other parameters of the oral medicine treatment group in the first month. With the exception of ESR, significant differences of others indicators between treatments were observed during the follow-up (Table 4).

## 4. Discussion

Earlier diagnosis and treatment are urgently required and effective in reducing disease burden of AS patients while both of them are usually impeded due to the seronegative character and the absence of hallmark. Thus a number of emerging researches are focusing on the new biomarkers and antibodies, which can not only contribute to the diagnosis, but also reflect disease activity and curative effect [5, 23], while the currently new biomarkers are still elusive due to the inconsistent results of different researches as well as lacking of multicenter studies. Therefore, only HLA-B27 and inflammatory marker CRP are widely accepted as routinely clinical biomarkers of AS. Detecting HLA-B27 is useful only if AS is strongly suspected due to lack of specificity [5, 24]. Moreover, only half of the AS patients with the evaluated CRP and earlier studies indicated the poor predictive value of CRP and the poor correlation with BASDAI [25, 26]; however, a study in 2010 showed a significant correlation between CRP and disease activity [27]. Thus, AS poses significant challenge to the quest for biomarkers to assess disease activity and monitor treatment response.

HMGB1 was initially recognized as a DNA-binding protein widespread in the mammal eukaryotic cell nucleus until the discovery that HMGB1 serves as a late inflammatory cytokine in the progress of sepsis and endotoxaemia in 1999 [28]. Extracellular HMGB1 acts as a proinflammatory mediator participating in the pathological processes of acute lung inflammation, transplant rejection, and ischemia-reperfusion injury [29–31] in addition to rheumatoid diseases such as systemic lupus erythematosus, rheumatoid arthritis, and Sjogren's syndrome [32–34]. However, little research has focused on the association between HMGB1 and AS. The literature thus far is inconsistent with respect to a correlation between serum HMGB1 level and disease status. Although two studies demonstrated that the level of HMGB1 was significantly higher than healthy controls, Chen et al. [17] suggested that HMGB1 might be a good laboratory candidate for the evaluation of disease severity and activity. However, Oktayoglu et al. [16] adhered to the opposite viewpoint. The contrasting results of these two studies may be due to the different inclusion criteria of AS patients and the fact that the number of cases was insufficient. In addition, these two studies did not observe the effects of different treatment strategies on HMGB1, as well as the correlation between HMGB1 and other important cytokines involved in the pathogenesis of AS.

In our present study, we collected a relatively large sample composed of 147 AS patients and 61 healthy controls that did not receive any drugs or treatment during the previous 3 months, thus avoiding the influence of medicine on HMGB1 levels. We found that the serum HMGB1 level was significantly higher in AS patients than in healthy controls (HMGB1: 106.81 ± 30.87 ng/mL versus 27.68 ± 17.95 ng/mL, *P* < 0.001). HMGB1 was also positively correlated with BASDAI, BASFI, ASDAS-ESR, ASDAS-CRP, CRP, and ESR. However, no correlation was detected between the serum HMGB1 level and HLA-B27 or with respect to extra-articular manifestations, including recurrent anterior uveitis and diarrhea. In addition, there were no statistically significant differences in the serum HMGB1 level between the AS patients with pathological changes involving hip joints and those without. Although CRP is considered as a laboratory indicator to reflect inflammation levels and disease activity we found that there were 13.61% patients who have high disease activity (BASDAI > 4) and severe clinical symptoms but with normal CRP values. Among patients with normal CRP, serum HMGB1 of the BASDAI > 4 group was significantly higher than in those with BASDAI ≤ 4, which indicated that HMGB1 may be a better laboratory index reflecting ongoing inflammatory disease, particularly in those cases with CRP in the normal range (Figure 1).

It is noteworthy that HMGB1 has a chemotactic effect on osteoblasts, monocytes, and endothelial cells, as well as the recruitment of mesenchymal stromal cells [9]. TNF-*α* can stimulate the activated monocyte-macrophage cells to secrete HMGB1, which then interacts with multiple receptors including RAGE, TLR2, and TLR4. HMGB1-mediated signaling can lead to the activation of nuclear factor *κ*B (NF-*κ*B) and the production of various cytokines such as IL-6 and TNF-*α*, which are involved in the pathogenesis of AS [35]. While the HMGB1-involved signal pathways which can participate in the pathogenesis of AS are still rather ambiguous, further in-depth study is needed.

The present study is the first long-term controlled trial focusing on variation in the HMGB1 level in AS patients receiving different treatment. Although several clinical trials have shown a relatively good response to TNF-*α* blockers as therapy for AS, there are individual differences between patients [36]. More sensitive laboratory indicators are needed for clinicians to predict the therapeutic effect of biological treatments. We observed that the TNF-*α* blockade group quickly achieved remission of joint symptoms and demonstrated an improved quality of life and work. Within this group, HMGB1 significantly decreased in the first month of treatment (20.21%, *P* < 0.05 versus baseline) and then maintained a relatively steady downward trend during treatment. This pattern was reflected in the disease activity indexes such as BASDAI, BASFI, ASDAS, CRP, and ESR. However, compared with baseline, the HMGB1 level of the oral treatment group did not have a statistical difference, while the other indicators had only slight fluctuations without a clear downward trend (Table 4). Further, there also existed the significant correlation between HMGB1 and AS related indicators (BASDAI, BASFI, ASDAS-ESR, ASDAS-CRP, ESR, and CRP) during the 6-month follow-up period. Therefore, to a certain extent, we speculate that the HMGB1 may be considered as an indicator to monitor the therapeutic effect.

Taken together, our results revealed an increase of HMGB1 levels in AS patients, but HMGB1 had no significant correlation with HLA-B27 and extra-articular manifestations. Although HMGB1 was not correlated with the radiographic destruction severity of involved joints, HMGB1 can reflect the activity and severity of AS disease and is a relatively good biomarker of the therapeutic effect to a certain extent. Further studies must be performed to clarify the pathogenetic role of HMGB1 in AS.

## Figures and Tables

**Table 1:** Baseline characteristics of 147 ankylosing spondylitis patients.

	AS	HC
Age (years)	30.28 ± 9.85	29.36 ± 5.85
Sex (male/female)	124/23	45/16
HMGB1 (ng/mL)^*∗∗*^	106.81 ± 30.87	27.68 ± 17.95
BASDAI	3.59 ± 1.71	
BASFI	2.48 ± 1.83	
ASDAS-ESR	2.89 ± 1.29	
ASDAS-CRP	2.97 ± 1.33	
ESR (mm/h)	32.93 ± 27.82	
CRP (mg/L)	24.97 ± 32.41	

AS: ankylosing spondylitis, HC: healthy control, BASDAI: Bath AS Disease Activity Index, BASFI: Bath AS Functional Index, ASDAS: AS Disease Activity Score, ESR: erythrocyte sedimentation rate, and CRP: C-reactive protein. Values are shown as mean ± SD. ^*∗∗*^ *P* < 0.01.

**Table 2:** Spearman's correlation analysis between serum HMGB1 and clinical parameters of the 147 AS patients, including the baseline and the follow-up time points.

	HMGB1	BASDAI	BASFI	ASDAS-ESR	ASDAS-CRP	CRP	ESR
HMGB1		0.304^*∗∗*^	0.184^*∗∗*^	0.275^*∗∗*^	0.251^*∗∗*^	0.132^*∗*^	0.162^*∗∗*^
BASDAI			0.635^*∗∗*^	0.668^*∗∗*^	0.678^*∗∗*^	0.364^*∗∗*^	0.385^*∗∗*^
BASFI				0.595^*∗∗*^	0.607^*∗∗*^	0.396^*∗∗*^	0.418^*∗∗*^
ASDAS-ESR					0.884^*∗∗*^	0.668^*∗∗*^	0.797^*∗∗*^
ASDAS-CRP						0.738^*∗∗*^	0.669^*∗∗*^
CRP							0.761^*∗*^

BASDAI: Bath AS Disease Activity Index, BASFI: Bath AS Functional Index, ASDAS: AS Disease Activity Score, ESR: erythrocyte sedimentation rate, and CRP: C-reactive protein. Values are shown as *r*. *r* is determined by Spearman's correlation analysis. ^*∗*^ *P* < 0.05. ^*∗∗*^ *P* < 0.01.

**Table 3:** Baseline characteristics of the two follow-up therapeutic AS groups.

	Anti-TNF	NSAIDs + SASP
*N*	25	16
Age (years)	28.48 ± 6.73	32.81 ± 9.40
HMGB1 (ng/mL)	101.76 ± 26.31	100.28 ± 28.83
BASDAI	4.07 ± 1.44	3.52 ± 0.96
BASFI	3.14 ± 1.94	2.77 ± 1.58
ASDAS-CRP	3.23 ± 0.72	2.82 ± 0.84
ASDAS-ESR	2.95 ± 0.79	2.90 ± 0.83
ESR (mm/h)	27.44 ± 29.22	28.13 ± 26.02
CRP (mg/L)	35.23 ± 28.10	26.22 ± 32.35

**Table 4:** Absolute changes in HMGB1, BASDAI, BASFI, ASDAS-ESR, ASDAS-CRP, ESR, and CRP in the two follow-up therapeutic AS groups.

	Anti-TNF	SASP + NSAIDs
HMGB1 change, mean (SD), ng/mL		
1 month	−20.57 (22.25)^ab^	1.52 (24.99)
3 months	−25.35 (23.70)^a^	−12.97 (19.89)^a^
6 months	−28.10 (28.68)^ab^	−3.89 (25.30)

BASDAI change, mean (SD)		
1 month	−1.87 (1.51)^ab^	−0.57 (0.51)^a^
3 months	−2.29 (1.61)^ab^	−1.06 (0.77)^a^
6 months	−2.57 (1.51)^ab^	−0.90 (1.16)^a^

BASFI change, mean (SD)		
1 month	−1.17 (1.56)^ab^	−0.15 (0.61)
3 months	−1.23 (1.65)^a^	−0.87 (1.00)^a^
6 months	−1.80 (1.69)^ab^	−0.74 (1.17)^a^

ASDAS-ESR change, mean (SD)		
1 month	−1.30 (1.07)^a^	−0.61 (0.59)^a^
3 months	−1.67 (0.86)^ab^	−0.82 (0.62)^a^
6 months	−1.70 (0.89)^ab^	−0.63 (0.69)^a^

ASDAS-CRP change, mean (SD)		
1 month	−1.57 (0.79)^ab^	−0.12 (0.81)
3 months	−1.72 (0.83)^ab^	−0.76 (1.00)^a^
6 months	−1.88 (0.63)^ab^	−0.58 (0.96)^a^

ESR change, mean (SD), mm/h		
1 month	−24.67 (24.61)^a^	−12.82 (19.22)
3 months	−29.47 (25.91)^a^	−14.15 (18.18)^a^
6 months	−28.96 (26.69)^a^	−13.94 (22.47)^a^

CRP change, mean (SD), mg/L		
1 month	−20.55 (12.99)^ab^	−4.25 (18.54)
3 months	−19.70 (25.99)^a^	−12.76 (25.57)
6 months	−24.23 (28.41)^a^	−8.04 (26.32)

^a^ *P* < 0.05 versus baseline.  ^b^ *P* < 0.05 versus SASP + NSAIDs.
